# Uniparental disomy in a population of 32,067 clinical exome trios

**DOI:** 10.1038/s41436-020-01092-8

**Published:** 2021-01-25

**Authors:** Julie Scuffins, Jennifer Keller-Ramey, Lindsay Dyer, Ganka Douglas, Rebecca Torene, Vladimir Gainullin, Jane Juusola, Jeanne Meck, Kyle Retterer

**Affiliations:** GeneDx, Gaithersburg, MD USA

## Abstract

**Purpose:**

Data on the clinical prevalence and spectrum of uniparental disomy (UPD) remain limited. Trio exome sequencing (ES) presents a comprehensive method for detection of UPD alongside sequence and copy-number variant analysis.

**Methods:**

We analyzed 32,067 ES trios referred for diagnostic testing to create a profile of UPD events and their disease associations. ES single-nucleotide polymorphism (SNP) and copy-number data were used to identify both whole-chromosome and segmental UPD and to categorize whole-chromosome results as isodisomy, heterodisomy, or mixed.

**Results:**

Ninety-nine whole-chromosome and 13 segmental UPD events were identified. Of these, 29 were associated with an imprinting disorder, and 16 were associated with a positive test result through homozygous sequence variants. Isodisomy was more commonly observed in large chromosomes along with a higher rate of homozygous pathogenic variants, while heterodisomy was more frequent in chromosomes associated with imprinting or trisomy mosaicism (14, 15, 16, 20, 22).

**Conclusion:**

Whole-chromosome UPD was observed in 0.31% of cases, resulting in a diagnostic finding in 0.14%. Only three UPD-positive cases had a diagnostic finding unrelated to the UPD. Thirteen UPD events were identified in cases with prior normal SNP chromosomal microarray results, demonstrating the additional diagnostic value of UPD detection by trio ES.

## INTRODUCTION

First proposed in 1980, inheritance of both copies of a chromosome from a single parent, or uniparental disomy (UPD), has been a known mechanism of disease for four decades.^1,2^ The American College of Medical Genetics and Genomics (ACMG) recently published a new set of points to consider regarding prenatal and postnatal testing for UPD, a tribute to the importance of this phenomenon as a cause of genetic disease.^3^ The clinical consequences of UPD depend on the chromosome involved. UPD of six autosomes are associated with disease through parent-of-origin effects. Known imprinting disorders include transient neonatal diabetes mellitus (OMIM 601410) due to paternal UPD of chromosome 6, Silver–Russell syndrome (OMIM 180860) due to maternal UPD of chromosomes 7 or 11, Beckwith–Wiedemann syndrome (OMIM 130650) resulting from paternal UPD of chromosome 11, Temple syndrome (OMIM 616222) and Kagami–Ogata syndrome (OMIM 608149) associated with maternal and paternal UPD of chromosome 14 respectively, Prader–Willi syndrome (OMIM 176270) and Angelman syndrome (OMIM 105830) due to maternal and paternal UPD of chromosome 15 respectively, and Mulchandani–Bhoj–Conlin syndrome (OMIM 617352) and pseudohypoparathyroidism (OMIM 103580) resulting from maternal and paternal UPD of chromosome 20 respectively.

However, parent-of-origin effects are not the only mechanism by which UPD may cause disease. A pathogenic variant in a recessive disease gene may be unmasked in a region of isodisomy, and therefore cause disease. In this way, UPD of any chromosome has the potential to result in a genetic disorder and a range of phenotypes. Finally, UPD may be an indication of a mosaic aneuploidy associated with disease. Uniparental disomy most commonly results from nondisjunction and subsequent trisomy rescue.^4–6^ While the rescue event can correct the chromosome number, a persistent mosaic trisomic cell line could contribute to the phenotype. If the trisomic cell line is below the limit of detection, UPD resulting from the rescue event may be identified instead. For example, maternal UPD16 has been reported in a patient subsequently determined also to have mosaic trisomy 16, which could account for the patient’s phenotype.^7^

In this study, we distinguished four types of UPD. Isodisomy occurs when two copies of a single homolog are inherited from one parent, resulting in homozygosity across the chromosome.^8,9^ Complete isodisomy may arise from trisomy rescue due to nondisjunction without recombination in gametogenesis, rescue of a monosomy due to nondisjunction in gametogenesis or postfertilization mitotic error, or from gamete complementation, a process in which one gamete contributes two copies of a chromosome while the other gamete contributes none of that chromosome.^6,10^ Heterodisomy refers to inheritance of both homologs from a single parent, and very little or no homozygosity is observed.^8,9^ We also distinguish a type of UPD in which large segments of both isodisomy and heterodisomy are identified across the chromosome, referred to here as “mixed UPD.” Complete heterodisomy and mixed UPD require transmission of two chromosomes from a single parent, thus UPD with heterodisomy arises either due to nondisjunction during gametogenesis and subsequent trisomy rescue or, less likely, gamete complementation.^6,10,11^ Finally, segmental UPD represents uniparental disomy that affects only a portion of a chromosome with the remainder showing biparental inheritance.^12^ Segmental UPD is thought to be the result of a postzygotic recombination between parental chromosomes, and may occur in the context of trisomy rescue. Deletion rescue is another type of rescue event that can result in segmental isodisomy and occurs when a chromosomal terminal deletion is stabilized by copying the missing segment from the opposite parental homolog.^6,11,13,14^ Most segmental UPD is isodisomy; however, segmental heterodisomy has been reported.^12^

Single-nucleotide polymorphism (SNP) microarray is commonly used as first-tier testing for individuals with neurodevelopmental disorders or multiple congenital anomalies; however, it is unlikely to detect complete or near-complete uniparental heterodisomy. Complete heterodisomy would be detected on SNP array only if trio genotype analysis was performed for all chromosomes, something that is not part of routine chromosomal microarray (CMA) analysis. A study of UPD detection by SNP microarray reported 10 of 30 confirmed UPD samples had no long contiguous stretches of homozygosity detected on the chromosome of interest, suggesting that up to one-third of whole-chromosome UPDs would not be detected by this method.^15^ Targeted methylation testing for UPD is also available when there is a suspected UPD-related diagnosis (most commonly UPDs 7, 11, 14, and 15), but may not be efficient for testing patients with less specific phenotypes. Recent studies have demonstrated detection of UPD through next-generation sequencing methods, and expanded our understanding of UPD as it occurs in general and clinical populations. A recent large population study using 916,712 parent–child pairs estimated instances of UPD to be 1:2,000 births (approximately 0.05%).^16^ A clinical exome sequencing (ES) study involving 4,912 trios and 29,723 singletons found UPD at a frequency of 0.2%,^17^ consistent with the expectation of a higher rate of UPD in the patient population. Exome trio testing can potentially identify UPD undetected by other methods. Here, we report a retrospective study of UPD in 32,067 trios referred for diagnostic ES, the largest clinical trio cohort reported to date, to enhance our understanding of UPD in the clinical population and to investigate the clinical utility of UPD testing by trio exome sequencing.

## MATERIALS AND METHODS

### Cohort composition

We analyzed 32,067 unique parent–child trios referred for clinical exome sequencing for a diverse set of phenotypic indications (Table S1). The median age of probands at the time of testing was 7.5 years. Samples were sequenced by Illumina HiSeq or NovaSeq 2×100 or 2×150 reads after hybridization capture using either Agilent Clinical Research Exome or IDT xGen Exome v1.0 baits, followed by variant calling as previously described.^18^ Positive findings, including imprinting, sequence, and copy-number variants, were confirmed by an orthogonal method and reported to the ordering provider.

### Identification of potential UPD regions

The detection method and algorithm (Figure S1) described below was developed internally in Perl, Python, and R program languages except where noted. For each trio, variant calls were subset to SNPs with a genotype quality ≥90 in all three. Variants with the potential to reduce reliability of observed inheritance patterns were excluded, including all indels for potential allele balance skewing and all variants within the MHC (chr6:28477797–33448354) and LRC (chr19:51403257–59118983) due to high rates of somatic variation. The sex chromosomes were also excluded. Remaining variants were categorized as Mendelian errors supportive of uniparental inheritance, as supportive of biparental inheritance, or as noninformative to the parent of origin by comparing the parent–child genotype combinations (Table S2). Each chromosome was then interrogated for the presence of Mendelian errors occurring without intervening variants that would support biparental inheritance. Regions of uninterrupted Mendelian errors ≥5 Mb in size and containing a minimum of 20 supporting variants were marked as possible regions of UPD. The length threshold was selected as both a common minimum threshold for reporting regions of homozygosity (ROH)^15^ and because it is large enough to provide sufficient SNP density from ES to call UPD across most of the genome.

### Screen for deleted regions

Apparent Mendelian errors can occur both due to the presence of UPD and due to a deletion of one allele making the resulting hemizygous variants appear as homozygous variant calls when called against a diploid model. Therefore, for each sample, copy-number variants were called using the read-depth method previously described, which reliably detects deletions smaller than the 5 Mb resolution of UPD detection.^19^ The copy number of each prospective UPD result was determined by cross-referencing copy-number variant data of the same region. Regions of Mendelian error determined by this method to represent deletions were removed from further UPD analysis.

### Annotation of UPD results

For each UPD-positive trio, we determined ROH using BCFtools *roh*,^20^ excluding indels. UPD results were then binned as either isodisomy, heterodisomy, or mixed UPD as follows: (1) isodisomy when the full UPD region matched detected ROH; (2) mixed UPD when part(s) of the UPD region, but not all, matched detected ROH; and (3) heterodisomy when no part of the UPD region matched a detected ROH. We excluded from the probands any ROH on the UPD chromosome that were also present as ROH in the parent of origin for UPD, since it is not possible to distinguish heterodisomy from isodisomy in these regions.

To determine the parent of origin for UPD, we assessed the inheritance patterns located within each UPD region. We assigned a region as maternal UPD if the proband contained only alleles inherited from the mother and was missing alleles inherited from the father, and paternal UPD if the proband contained only alleles inherited from the father and was missing alleles inherited from the mother.

Finally, we assessed whether each result spanned the whole chromosome or only a segment. For segmental UPD, the boundaries of the result were defined as the coordinates of the first and last Mendelian errors identified, or the terminus if no informative variants were identified between the final Mendelian error and the end of the chromosome.

## RESULTS

**Fig. 1:**
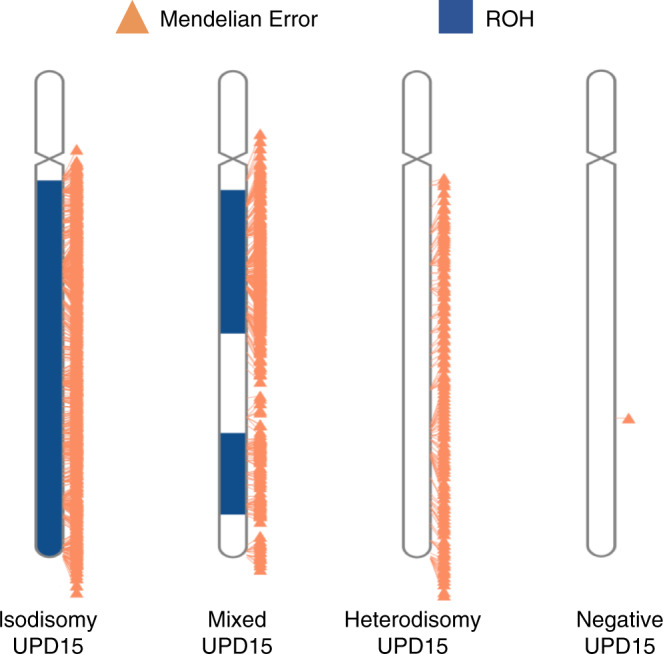
Locations of Mendelian errors and regions of homozygosity (ROH) on chromosome 15 for four probands, plotted with RIdeogram^27^. Each triangle is a single Mendelian error with a line connecting it to its position on the chromosome (note that there are no protein-coding genes on 15p). Three samples are shown who were identified as having isodisomy, mixed uniparental disomy (UPD), and heterodisomy of chr15. For these samples, Mendelian errors were identified across all targeted regions of the chromosome, irrespective of the amount of ROH identified in the sample. Conversely, in a sample negative for UPD15 there is a lack of Mendelian errors indicating biparental inheritance. Only the presence of Mendelian errors distinguishes the chromosome with heterodisomy from the negative sample, demonstrating that ROH detection alone would not have discovered this instance of UPD15.

Of 32,067 trios, we identified 99 events of whole-chromosome UPD and 13 events of segmental UPD (Table 1). Representative examples of isodisomy, mixed, heterodisomy, and negative UPD15 are shown in Fig. 1.

**Table 1:** The number of cases and parent of origin for each type of uniparental disomy (UPD) identified.

	Isodisomy	Heterodisomy	Mixed	Segmental
Maternal	14	13	42	3
Paternal	25	2	3	10
Total	39	15	45	13

### Whole-chromosome UPD

**Fig. 2:**
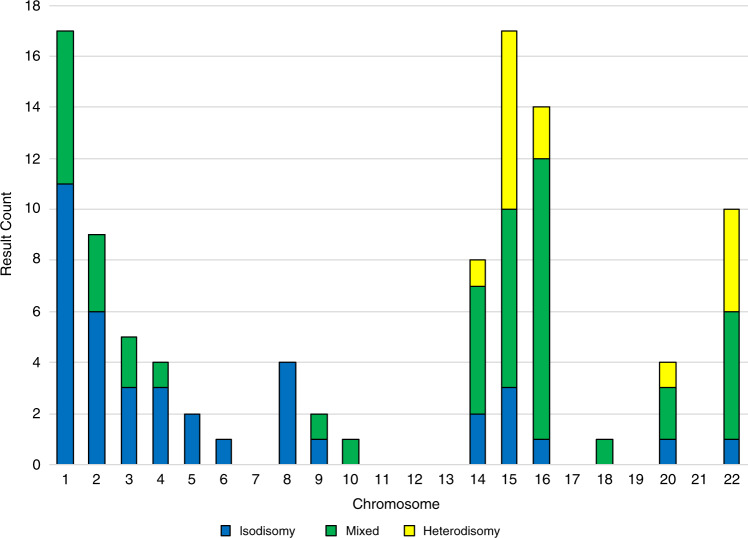
Uniparental disomy (UPD) type and chromosome involved for 99 cases of whole-chromosome UPD. Each vertical bar represents the total number of UPD results observed for that chromosome, broken down as complete isodisomy, mixed UPD, and complete heterodisomy.

Of the 99 events of whole-chromosome UPD occurring on 15 different chromosomes, chromosomes 1 and 15 (17 observations each), as well as 16, 2, 22, and 14, were most commonly involved (Fig. 2). The overall rate of whole-chromosome UPD within the cohort was 3 in 1,000; this was significantly higher than the rate of 1:2,000 previously reported within the general population (Fisher’s exact, *p* = 2.2E-68).^16^ No samples were observed with UPD of more than one chromosome; however, four samples within the whole-chromosome UPD set (4.0%) also exhibited aneuploidy involving either chromosome X or Y.

Of the whole-chromosome events, we observed 45 with mixed UPD, 39 with complete isodisomy, and 15 with complete heterodisomy. Complete isodisomy was observed more commonly on chromosomes 1 and 2 (11 and 6 events respectively), while complete heterodisomy was observed only in chromosomes 15, 22, 16, 14, and 20 (7, 4, 2, 1, and 1 events, respectively).

A median of 50,846 variants per sample met the established filtering criteria (Table S3), with a median of 0 and average of 1.55 Mendelian errors identified across all non-UPD chromosomes. On the chromosomes positive for whole-chromosome UPD, we observed that Mendelian errors made up a smaller proportion of high-quality trio SNPs in complete heterodisomy chromosomes (median 6.09%) than complete isodisomy chromosomes (median 19.71%, Wilcox test, *p* = 1.71E-8), but never fell below 4.5% of the total variant sites (Table S4).

### Whole-chromosome UPD and association with test outcome

**Fig. 3:**
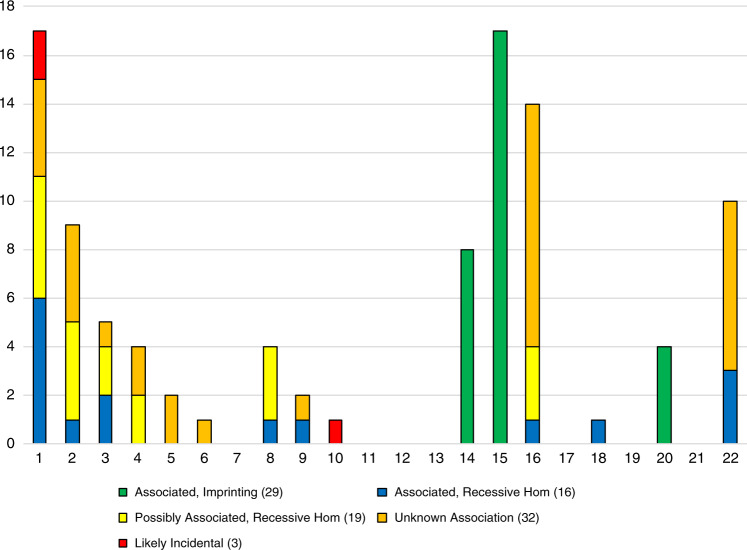
Association of 99 instances of whole-chromosome uniparental disomy (UPD) with patient phenotype. A UPD result was considered associated with the patient phenotype if it overlapped with a known imprinting region or with pathogenic or likely pathogenic bi-allelic sequence variants in a gene correlating with the phenotype. UPD was considered possibly associated if sequence variants were identified in a gene with phenotypic overlap but not able to be classified as likely pathogenic. The UPD was considered as likely incidental if one or more variants were identified on a chromosome other than the UPD chromosome which correlated with the patient phenotype. Finally, if no overlap with an imprinting region was present and no variants were identified which correlated with the phenotype, the association of UPD to the patient phenotype was considered unknown.

Forty-five of 99 whole-chromosome UPD results were directly or indirectly diagnostic (45%, Fig. 3). UPD was directly diagnostic in 29 cases (29%) for imprinting disorders of chromosomes 14, 15, and 20 that correlated with the patient phenotype. Of these results, 9/29 (31%) were comprised of complete heterodisomy (seven from chr15, one each from chr14 and chr20). An additional 16 indirectly diagnostic UPD findings revealed pathogenic or likely pathogenic biallelic sequence variants, as classified by ACMG criteria,^21^ or copy-number variants that were associated with the patient phenotype (Table S5).

Of the remaining 54 UPD-positive cases, 3 had a causative variant on a chromosome other than the UPD chromosome, suggesting that those instances of UPD, two findings of UPD1 and one finding of UPD10, were possibly benign or secondary findings unrelated to the indication for testing. In 19 cases, homozygous variants of unknown significance or variants in candidate genes were identified on the UPD chromosome and reported. However, in 32 individuals, no variants could be identified to explain the patient phenotype either on the UPD chromosome or any other, and only the presence of UPD was reported as a finding of unknown significance.

### Segmental UPD and association with test outcome

We identified 13 occurrences of segmental UPD, including one observation each on chromosomes 2, 6, 7, 8, 13, 14, 15, 16, 18, 19, and 21, and two observations on chromosome 1 each unique in size and location. Four of these chromosomes (7, 13, 19, and 21) were not observed in the whole-chromosome UPD cohort. Sizes of the segmental UPDs ranged from 5 to 93 Mb with a median size of 16 Mb. All 13 were consistent with isodisomy based on overlap with detected ROH, with the remainder of the chromosomes showing biparental inheritance. Additionally, all 13 segmental events were associated with one of the terminal ends of the chromosome (Table S6), and none crossed the centromere, although three extended through a full chromosome arm. In contrast, large ROH associated with heterozygous deletions were interstitially located 45.7% of the time (16/35 occurrences).

Only 3 of 13 segmental UPD results (23.05%) were able to be associated with the patient’s phenotype: one due to overlap with a known imprinting region, one due to unmasking of a homozygous recessive variant, and one as part of a compound structural rearrangement. Of the remaining ten cases, no other causative variants were identified either on the UPD chromosome or any other.

### Parent-of-origin analysis shows possible influence of monosomy rescue

Of the whole-chromosome UPD events, 69 were maternally inherited and 30 were paternally inherited, showing a significantly higher rate of maternal UPD (binomial test, *p* = 3.24E-5). The parent of origin varied by the type of UPD present (Table 1). For complete heterodisomy and mixed UPD, the parent of origin was maternal in 55/60 events (91.6%). However, for complete isodisomy, the parent of origin was maternal in just 14/39 events (35.9%).

The parent in whom nondisjunction occurred cannot be conclusively determined for instances of complete isodisomy, as isodisomy could arise from multiple mechanisms: trisomy rescue after nondisjunction in the parent of-origin, monosomy rescue after nondisjunction in the opposite parent, or monosomy rescue due to nondisjunction in gametogenesis. Therefore, we selected only the 60 instances of heterodisomy or mixed UPD, where the timing and parent of origin for the UPD is unambiguous, to review the effect of parental age on the incidence of UPD. For 30,917 trios where parental age at birth could be determined, we observed that the average maternal age of maternal UPD cases (37.35 years) was significantly higher than the non-UPD population (30.33, Wilcox test, *p* = 1.07E-06). The average age of paternal UPD cases was not evaluated due to the small sample size (*N* = 4).

### Review of prior testing shows higher yield of trio exomes

**Fig. 4:**
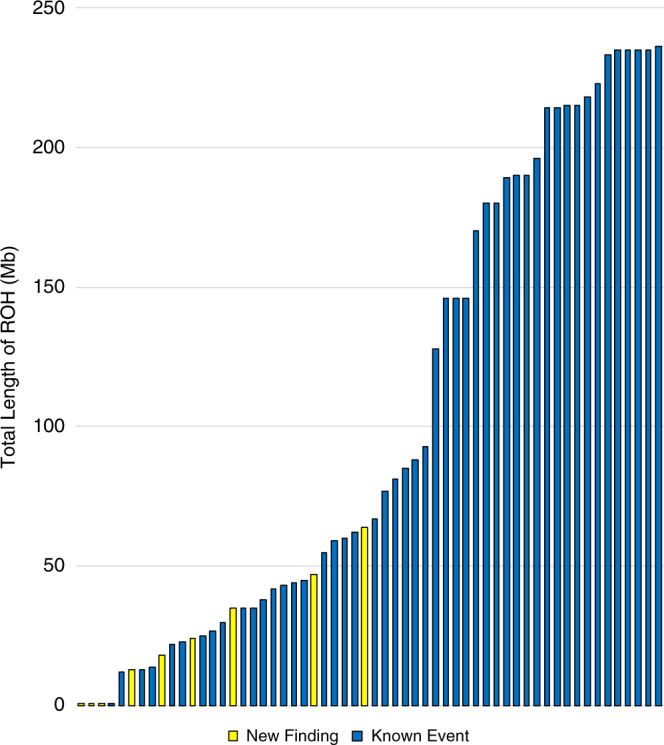
The combined length of regions of homozygosity (ROH) for whole-chromosome uniparental disomy (UPD) events in 58 probands for whom prior testing was potentially capable of detecting UPD. In instances of isodisomy, ROH spanned the full length of the chromosome. However, in Mixed UPD, multiple ROH were identified which were separated by regions of heterozygosity.

To assess the utility of our method, we performed a review of any prior testing noted within the available medical records of the 99 probands with whole-chromosome UPD, to determine if the presence of UPD was a known finding. We identified 58/99 probands who had received a genetic test with the potential to detect UPD, either a SNP microarray, targeted methylation testing, or, in one case, a prior exome test. Of those 58, UPD was a known or suspected finding in 49 patients based on the results of the prior test and was a new finding for 9 patients (15.5% of those with relevant prior testing). For the 9 patients with new UPD findings, 3 results were consistent with complete heterodisomy and 6 results consistent with mixed UPD. The total length of ROH in the new findings (M = 23.3 Mb, SD = 22.7) was significantly smaller than the average ROH in previously detected results (M = 117.2 Mb, SD = 82.8, *t*-test, *p* = 0.001277, Fig. 4). Importantly, the majority of these new findings (6 of 9), were diagnostic and related to the patient’s phenotype through imprinting disorders (specifically chromosomes 14 or 15). Two additional findings were associated with biallelic recessive variants, and one had unknown association to the phenotype.

We also reviewed available prior testing information for the 31,968 trios that we found negative for UPD, to look for instances of previously known UPD that had not been detected via our method. Medical history data was provided for 24,453 trios, including 3,407 tests mentioning a prior array and 500 tests mentioning prior methylation testing of one or more chromosomes. We were unable to identify any instances of confirmed UPD. However, we did identify three tests where UPD was suspected by the ordering physician due to known ROH, and an additional four tests where a prior array had detected large ROH (>5 Mb) on a single chromosome, raising suspicion for isodisomy. Review of the exome sequencing data for these patients identified the previously known ROH, but found no Mendelian errors present within the ROH regions or suspected UPD chromosome, suggesting that these ROH represent identity by descent rather than UPD. Overall, our review of prior testing supports high confidence in our method for detecting UPD.

## DISCUSSION

UPD was recently estimated to occur at a frequency of 1:2,000 births in a large study involving trios from a general population database.^16^ In contrast, a study of UPD in trios from a clinical cohort with suspected genetic disorders identified UPD events at a rate of approximately 2:1,000 (0.2%).^17^ The frequency of whole-chromosome UPD in our cohort consisting primarily of young patients with neurodevelopmental disorders was 3:1,000. We suspect this difference is driven by the inclusion of nontrios and the apparent inability to detect heterodisomy in the earlier study.

While we expect to have enrichment for potentially pathogenic UPD in our clinical cohort relative to the general population, a large number of our patients had previous genetic testing including microarray and methylation analysis, suggesting that our cohort should be depleted for UPD disorders that are detected by these methods. Thus, the true frequency of UPD in the patient population may be higher than measured in this study.

The majority of the UPD identified in our cohort involved chromosomes or regions not currently known to have parent-of-origin effects. Based on the previously reported rate of UPD in the general population^16^ (1:2,000), we would expect about 16 instances of “benign” UPD in our cohort. However, in 61 cases neither the identified UPD (51 whole-chromosome UPD, 10 segmental UPD) nor a variant on a non-UPD chromosome could be definitively linked to the patient phenotype. These cases likely represent a combination of benign UPD and cases in which the disease association of the UPD variant is not yet known. Discovery of novel disease genes or imprinting regions may enable additional cases to be solved, as well as follow-up testing capable of detecting mosaicism for aneuploid cell lines. While it is possible for ES to detect mosaicism of both single-nucleotide variants and copy-number variants including whole-chromosome aneuploidies, this ability is sample specific and the rate and limits of detection for mosaicism in ES have not been established. Furthermore, the strict filter on variant genotype quality set in this study likely inhibited our ability to detect instances of mosaic UPD. Since mosaicism for segmental, whole-chromosome, or genome-wide UPD is a known mechanism of disease, this presents an area for future improvement. It is possible that the true rate of UPD in our population is higher than reported due to undetected mosaicism.

Sixty-three of the 99 UPD-positive patients in our cohort had previous genetic testing including methods capable of detecting evidence of UPD including SNP array (59 cases), methylation studies (3 cases, one also tested by SNP array), or previous exome testing (1 case). Interestingly, ES identified a new UPD finding in 9 cases in which previous testing capable of identifying UPD had been performed. Clinically relevant findings were associated with these UPDs in all but one of the cases (six were positive for imprinting disorders and two were positive for homozygous recessive variants). In one of these cases, SNP array identified ROH on chromosome 15, but subsequent methylation testing returned as normal. In another, no array was performed, but relevant methylation testing was reportedly normal. Exome testing identified maternal UPD15, subsequently confirmed by methylation-specific multiplex ligation-dependent probe amplification (MS-MLPA), in both cases. One additional case had a SNP array that identified significant ROH on three chromosomes, so UPD was not strongly suspected but was identified on one of the three chromosomes by exome. Of the remaining cases, all with reportedly normal SNP array, two were identified to have full chromosome heterodisomy and four had mixed UPD (largest ROH ranging in size from approximately 13 to 30 Mb). SNP array is not expected to detect full heterodisomy, but it is unclear why the mixed UPD cases, three of which involved known imprinted chromosomes, were not reported on the SNP array.

We were surprised by the lack of UPD affecting chromosomes 6, 7, and 11 in our cohort. One explanation may be that disorders with unique hallmarks and consistent phenotypes between patients may be more readily identified in the clinic and sent for targeted testing, while disorders with a broad phenotypic spectrum may be more likely to be sent for exome testing. Additionally, imprinting-associated disorders will be underrepresented in our data set where mosaicism for UPD is a primary mechanism of disease due to the limitations of our method for detecting mosaic events. Notably, mosaic paternal UPD of chr11p15.5 is a common mechanism of disease for Beckwith–Wiedemann syndrome.^22^ Finally, the absence of these events in our cohort may simply reflect the relative rarity of these UPD disorders in our referral population.

For example, paternal UPD6 causes transient neonatal diabetes mellitus (TNDM) in an estimated 1 in 400,000 to 1 in 500,000 newborns.^23^ In our cohort of 32,067 trios, we identified a single UPD6 case with maternal origin, which is not associated with disease. It may be that our cohort is not large enough to necessarily include a paternal UPD6 case. It is also likely that patients with TNDM are reliably recognized in the clinic and targeted testing methods are chosen, depleting these cases from the exome testing cohort.

Complete isodisomy was the predominant UPD type observed in largest chromosomes (1–4), while all instances of complete heterodisomy were observed on only five chromosomes: 14, 15, 16, 20, and 22. A similar enrichment of isodisomy in the larger chromosomes was observed in Yauy et al., although no full heterodisomy was reported in their set.^17^ Paternal UPD are enriched for isodisomy, consistent with findings in previous studies.^9,16^ The average maternal age was increased for the UPD-positive cases relative to non-UPD cases. Maternal nondisjunction is known to be significantly more frequent than paternal nondisjunction, increasing with age.^9,24^ The increased rate of paternal isodisomy described here is likely the result of maternal nondisjunction and monosomy rescue involving the paternal chromosome.^9^ Isodisomy of the larger chromosomes may suggest that monosomy is a more frequent underlying mechanism for UPD of the larger chromosomes than trisomy. However, these data are also consistent with enrichment for isodisomy in nonimprinted chromosomes, where pathogenicity is associated with homozygosity, and enrichment for full heterodisomy in the imprinted chromosome set.

The utility of ES or, implicitly, genome sequencing extends beyond the identification of sequence variants. Deeper analysis of the data may be used to identify UPD. In many cases, chromosomal microarray is the first-tier test^25^ and most have a SNP component that permits identification of ROH indicative of UPD. Trio ES with UPD analysis can complement or potentially replace these tests by identifying whole-chromosome heterodisomy, distinguishing segmental UPD from whole-chromosome UPD, and detecting smaller UPD regions than may be found by other testing. ES is increasingly recommended and used as a first-tier diagnostic test for neurodevelopmental disorders,^26^ with parent–child trios making up the largest portion of tests submitted in our review period (59.89%). The ability to detect ROH, UPD, and underlying causative sequence variants provides additional support for that position, and supports the value of UPD detection in ES for diagnosis of genetic disorders and potentially for new disease discovery.

## Supplementary information

Supplemental Data

Supplementary Table S1

Supplementary Table S4

## Data Availability

All data supporting this study is included with the paper with the exception of individual alignment and variant call files, to comply with Health Insurance Portability and Accountability Act of 1996 (HIPAA) protections and the consent for aggregate, de-identified research. All sequence variants have been submitted to ClinVar (https://www.ncbi.nlm.nih.gov/clinvar/) as listed in Supplementary Table S5 (ClinVar accessions RCV000356077, VCV000372895, VCV000008953, VCV000014188, VCV000234471, VCV000372820, VCV000432173, VCV000977651, VCV000524013, VCV000546181, VCV000235732, VCV000595193, VCV000431812, VCV000005681, VCV000068165).

## Code availability

The software code used to identify the UPD findings presented here is not publicly available as it is integrated into the proprietary GeneDx commercial laboratory analysis platform and does not function as standalone software. However, relevant sections of the code are available upon reasonable request from the corresponding author.
