# Supplementary material for: Uniparental disomy in a population of 32,067 clinical exome trios
Source: Genet Med. 2021 Jan 25;23(6):1101–7. doi: 10.1038/s41436-020-01092-8 (PMC8187148; doi:10.1038/s41436-020-01092-8)
Supplement: Supplementary file 1 — Supplemental Data [file 41436_2020_1092_MOESM1_ESM.pdf]

Supplemental Data

Supplemental Figure S1. Schematic diagram of the algorithm for identification of high confidence stretches of Mendelian error, filtering by size and observed copy number, and ultimate identification of UPD and disomy type.

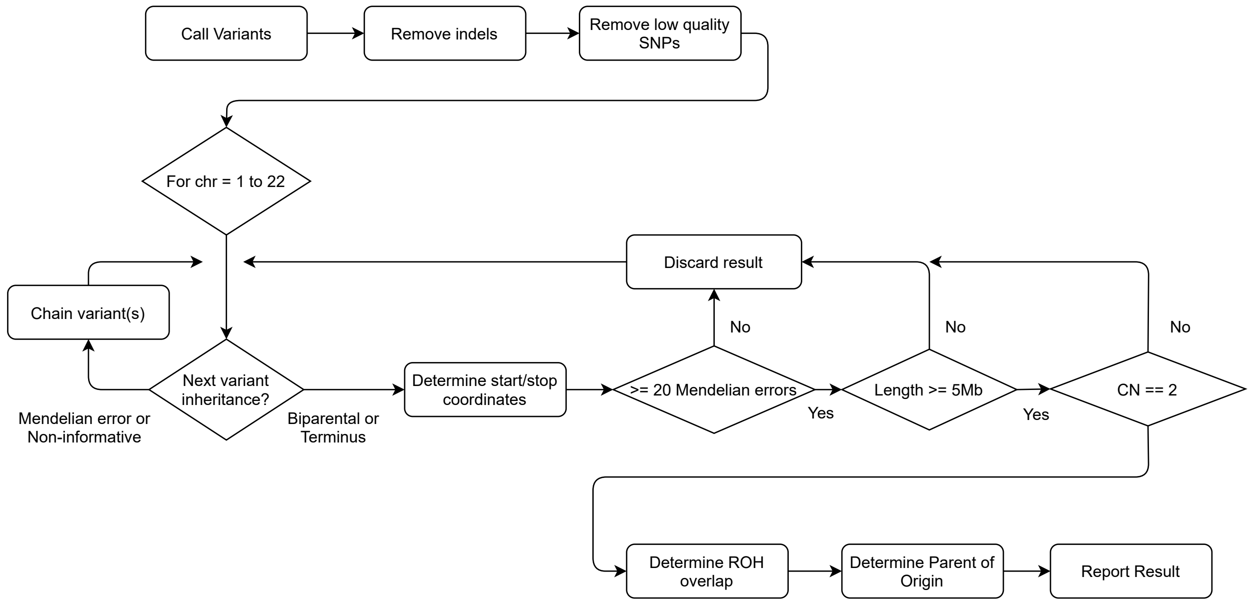

Table S1 (Excel file). Proband phenotypes within the UPD cohort overlapped a median of 16 HPO terms based on provided clinical records. 50 phenotypes were observed in > 5% of the probands.

Table S2. Trio genotype combinations supporting either uniparental or biparental inheritance. Combinations not appearing below would be non-informative to the parent of origin.

Class	***Child Genotype***	***Parental Genotype 1***	***Parental Genotype 2***
Mendelian error	hom	hom	wt
Mendelian error	wt	wt	hom
Biparental	het	hom	wt

Table S3. SNVs meeting filtering criteria per chromosome. A median of 50,846 total variants per sample were analyzed.

Chromosome	*Median High-Quality Variant Sites*
chr1	7150
chr2	4293
chr3	3502
chr4	2420
chr5	2627
chr6	2672
chr7	3477
chr8	2226
chr9	2903
chr10	3151
chr11	4439
chr12	3347
chr13	1103
chr14	1935
chr15	2411
chr16	3454
chr17	4562
chr18	1014
chr19	4560
chr20	1711
chr21	1107
chr22	1839

Table S4 (Excel file). Out of all whole chromosome UPD events which were categorized as complete heterodisomy or complete isodisomy, fewer Mendelian errors were detected on average in chromosomes with heterodisomy and made up a smaller proportion of high-quality variant sites.

Table S5. Homozygous variants identified on UPD chromosomes as causatively associated with a patient's provided phenotype.

ClinVar Accession	Location (hg19)	Gene	HGVS
RCV000356077	chr1:40555163	*PPT1*	NM_000310.3:c.455delG
VCV000372895	chr1: 43902925-43902927	*SZT2*	NM_015284.4:c.5949_5951del
VCV000008953	chr1:53668099	*CPT2*	NM_000098.3:c.338C>T
VCV000014188	chr1:161277049	*MPZ*	NM_000530.8:c.233C>T
VCV000234471	chr1:197070257	*ASPM*	NM_018136.5:c.8124T>G
VCV000372820	chr1:240492679	*FMN2*	NM_020066.5:c.4348C>T
VCV000432173	chr2:210836937	*UNC80*	NM_032504.1:c.8071G>T
VCV000977651	chr2:217300074	*SMARCAL1*	NM_014140.4:c.1499G>A
VCV000524013	chr3:123166964-123166977	*ADCY5*	NM_183357.2:c.402_415CGGCGGCGGCGGCT
VCV000546181	chr3:186980510	*MASP1*	NM_139125.3:c.238-2A>T
VCV000235732	chr8:97172848	*GDF6*	NM_001001557.4:c.73C>T
In progress	chr16:28490044-28496049	*CLN3*	NC_000016.9:g.28490044_28496049[4]
VCV000595193	chr18:55322503	*ATP8B1*	NM_005603.6:c.2854C>T
VCV000431812	chr22:43026971	*CYB5R3*	NM_000398.7:c.250C>T
VCV000005681	chr22:50962423	*NCAPH2*	NM_005138.2:c.418G>A
VCV000068165	chr22:51064623	*ARSA*	NM_000487.6:c.938G>A

Table S6. Chromosomal location of segmental Mendelian error events which were determined to represent either deletions or UPD.

	Deletion	*Segmental UPD*
Terminal	16	13
Interstitial	19	0
Total	35	13
